# Iodine deficiency and associated factors among school children: a cross-sectional study in Ethiopia

**DOI:** 10.1186/s13690-016-0158-4

**Published:** 2016-10-31

**Authors:** Sintayehu Hailu, Mamo Wubshet, Haile Woldie, Amare Tariku

**Affiliations:** 1 Department of Public Health, College of Medicine and Health Sciences, Madawalabu University, Goba, Ethiopia; 2 Department of Environmental and Occupational Health and Safety, College of Medicine and Health Sciences, Institute of Public Health, University of Gondar, Gondar, Ethiopia; 3 Department of Human Nutrition, Institute of Public Health, College of Medicine and Health Sciences, University of Gondar, Gondar, Ethiopia

**Keywords:** Low urine iodine level, Goiter, School children, Southeast Ethiopia

## Abstract

**Background:**

Iodine deficiency remains a public health problem in the world. It is the leading cause of preventable mental retardation and brain damage worldwide. Though 12 million school age children are at risk of developing iodine deficiency, there is a scarcity of literature showing the magnitude of iodine deficiency in Ethiopia. Therefore, this study aimed to determine the prevalence and associated factors of iodine deficiency among school children in Robe District, southeast Ethiopia.

**Methods:**

A school based cross–sectional study was conducted from February to June, 2015. A structured interviewer-administered questionnaire was used to collect data. A systematic random sampling technique was employed to select 422 children. A multivariate logistic regression analysis was carried out to identify factors associated with iodine deficiency. In the multivariate analysis, variables with a *P*-value of <0.05 were considered statistically significant.

**Results:**

A total of 393 school children participated in the study. The median urinary iodine level was 78 μg/l. About 57 and 43.5 % of the children were found with low urinary iodine level and goiter, respectively. Only 29 % of the households utilized adequately iodized salt. The result of the multivariate analysis revealed that the odds of iodine deficiency were higher among female [AOR = 2.23; 95 % CI: 1.54, 3.55] and older (10–12 years) [AOR = 2.21; 95 % CI: 1.44, 3.42] children.

**Conclusion:**

In this community, the prevalence of goiter and low urine iodine level is high. Thus, iodine deficiency exists as severe public health problem. In addition, there is a low utilization of iodized salt in the setting. Therefore, it is crucial to intensify efforts in the implementation of iodized salt. Moreover, attention should be given to school children to address ID.

## Background

Iodine is an essential micronutrient for supporting different physiological functions, however its deficiency is associated with a wider range of health problems [1–3]. Iodine Deficiency (ID) is considered as the most common preventable cause of mental retardation [4], and it results in decreased resistance against infections, poor school performance, and lack of physical strength of the child [5]. Moreover, previous reports claimed that ID causes 25 % of the Disability Adjusted Life Years (DALY’s) occurring in Africa [6]. As a result, it is found to significantly affect the socio-economic development of the nation at large [7]. Compared to other population segments, pregnant mothers and school children are the most vulnerable groups for ID [8].

ID is a global public health problem which is endemic in the mountainous regions of Europe, Asia, South and Central America and Eastern Africa [9]. Globally, 241 million school children are suffering from Iodine Deficiency Disorders (IDD), while more than 1.5 billion people are at risk of ID [10]. Likewise, IDD is observed among 57 million African children [10–12]. In Africa, in spite of its public health importance, the high burden of communicable diseases, socio-economic crisis, and political instabilities in the past have made the elimination of ID more challenging [13]. In Ethiopia, ID has remained a public health problem among school children for many decades [14–18], and the prevalence rate has reached 59.1 % in Senbo District, southwest Ethiopia [19]. Also, it causes 37.3 cretins, 33.4 miscarriage, and 47.5 still births and neonatal deaths per 1000 live births [20].

ID is mainly caused by low iodine content in the diet, arising from low iodine levels in the soil, water, or crops [21]. In addition, the consumption of goiterogenic substance containing food items, like cassava and millet [22], and co-existing micronutrient deficiencies (iron, selenium and vitamin A deficiency) [23], poor household socio-economic status, low maternal education, the unavailability of latrine, advanced age and sex of the child [24–27] are some of the factors associated with ID.

Cognizant of the problem, the Government of Ethiopia has planned to eradicate ID and to achieve the utilization of adequately iodized salt to 90 % by the year 2015 [28]. Furthermore, the Federal Ministry of Health designed a National Nutrition Program and micronutrient guideline, and endorsed a proclamation for ensuring the availability of iodized salt [28, 29], though significant changes have not been attained [30]. Thus, investigating the burden of ID have a paramount significance to evaluate the progress of the current interventions, however literature is dearth, particularly in the study area. Therefore to fill the knowledge gap, this study aimed to estimate the prevalence and associated factors of ID among school children (6–12 years) in Robe District, southeast Ethiopia.

## Methods

### Study setting

A school based cross-sectional study was conducted in Robe District from February to June, 2015. The district is found in Oromia Regional State, southeast Ethiopia, 430 km from the capital city of Ethiopia, Addis Ababa. Robe District is found between 2510 and 2800 m above sea level, and gets an average rainfall of 800–900 ml twice a year. Administratively the district is structured into a total of three kebeles *(smallest administrative unit in Ethiopia)*. According to the 2015 Robe District Finance and Economic Development Report, a total of 73,386 people live in the area, and women constitute 47.5 % (37,043) of the total population. The district has seven government primary schools.

### Sample size and sampling procedure

The required sample size for the study was determined using a single population proportion formula by considering the following assumptions: 50 % expected prevalence of ID (low urinary iodine level), at a 95 % confidence level, and a 5 % margin of error (d). Finally, a sample size of 422 was obtained after adding a 10 % non–response rate. A systematic sampling technique was employed to select the study participants. The total number of children going to each government primary school was obtained from the school director, and was divided by the total sample size to get the sampling fraction (K^th^ = N/n). Moreover, the total number of eligible children selected from each school was proportionate-to-population size.

### Data collection tools and procedures

A structured interviewer-administered questionnaire was used to collect data. The questionnaire was constructed in three major sections, socio-demographic and economic variables, and health and feeding pattern related characteristics. A total of seven data collectors (2 health officers, 2 clinical nurses and 2 laboratory technicians) and one supervisor (health officer) were recruited and participated in the study. Only urine sample was taken from each child, and a home visit was made to gather the rest of the information from the mother/care giver of the child.

To ensure consistency, the English version of the questionnaire was translated into Afan-Oromo (the native language of the study area) and then back translated to English by English language and public health experts. Two days of training was given to data collectors and supervisors. The training mainly focused on equipping the trainees on the objective of the study, technique of interview, collection of samples, and maintaining of ethical issues. The data collection tool was pretested on 5 % of the study subjects out of the selected schools. During the pretest, the acceptability and applicability of the procedures and tools were evaluated. All questioners were regularly checked for completeness, clarity, and consistency by the field supervisor. Furthermore, the investigators coordinated the overall data collection activities.

### Measurements

In cases of individuals with normal physiological functions, about 90 % of body iodine is excreted through urine; as a result estimating the median urine iodine level is supposed to be an important indicator of the burden of ID for the entire population [5]. Also, it has been considered as the most reliable measure of the current iodine intake [13]. Mindful of the established facts in determining the urinary iodine level, we took 5–10 ml of urine sample from each child, and collected it in a properly labeled and sterilized screw caped plastic bottle. The sample was kept at 4 °C in a refrigerator with all precautionary measures until analysis. The urinary iodine level was analyzed based on the ammonium per sulfate method [31] in the Ethiopian Health and Nutrition Research Institute (EHNRI), and the result was expressed in micrograms of iodine per 100 ml of urine. Finally, the iodine status of the child was interpreted as severe ID if the urine iodine level was <20 μg/l, while the participant was deemed to have a moderate and mild ID if their urine iodine level ranged from 20–49.9 μg/l and 50–99.9 μg/l, respectively. Additionally, a child was said to have adequate or sufficient iodine status if its iodine level was between 100 and 199.9 μg/l, but having a urinary iodine level of 200–300 μg/l and >300 μg/l was declared as an excessive level of iodine in the body [5].

The inspection and palpation of the thyroid gland provides information about the size (enlargement), consistency, and surface of the thyroid [8]. As a result, physical examination was performed by trained health officers to determine the size of the thyroid gland. Goiter was clinically defined according to the WHO criteria: grade-0 (no palpable goiter), grade-1 (palpable and visible goiter with extended neck), and grade-2 (visible goiter with the head in normal position) [10].

Regarding the determination of salt iodine content, the data collectors took the sample of salt used for cooking at the study participants home. Kit on spot was used to determine the iodine content of the salt, and it contains a small white cup, two test solution ampoules of 10 ml, and a recheck solution ampoule of 10 ml. In addition, it has a color chart indicators for iodine content showing a 0 ppm, less than 15 ppm, and ≥15 ppm. The small cup in the kit was filled with salt and made the cup surface flat. The two drops of properly shacked test solutions were added on the surface of the salt by piercing the white ampoule with a pin and gently squeezing the ampoule. The iodine content of salt was determined after 1 min by comparing the color developed on the salt with the chart color. If no color appears after 1 min, 5 drops of the recheck solution were added in the red ampoule containing a fresh salt sample spot, and then 2 drops of test solution on the same spot. Finally, the color of the sample was compared with the chart color and the salt iodine content was ascertained using this color result [10].

The standardized Individual Dietary Diversity Score tool tailored from the Food and Nutrition Technical Assistance (FANTA 2011) was used to qualitatively assess the dietary intake of children. The tool contained nine food groups, and it was aimed to reflect the micronutrient adequacy of the diet [32]. Mothers/guardians were requested to list the food consumed by the children in the previous 24 h preceeding the date of survey, and the reported food items were categorized into the nine food groups. The final DDS containg a maximum of nine scores was classified into three categories as poor, medium, and high dietary diversity when the child was served with a diet containing ≤3, 4–5, and ≥6 food groups, respectively [32].

### Statistical analysis

Data were entered into Epi-info version 3.5.3 and analyzed using Statistical Package for Social Sciences (SPSS) version 20. Descriptive statistics, including frequencies and proportions were used to summarize variables. A bivariable analysis was carried out to see the crude effect of each independent variable on ID (urinary iodine level of <100 μg/l). Variables with a *P*-values of <0.2 were entered into the multivariable logistic regression analysis. Both Crude Odds Ratio (COR) and Adjusted Odds Ratio (AOR) with a corresponding 95 % Confidence Interval (CI) were computed to show the strength of the association. In the adjusted analysis, a *P*-value of <0.05 was used to declare statistical significance.

## Results

A total of 393 children participated in the study giving a response rate of 93.1 %. The mean (±Standard Deviation, SD) age of the children was 9.15 (±1.6) years and nearly two-third (46.7 %) were aged 6–9 years. About two-third (72.0 %) of the children had more than five family size members. Almost all of the mothers (90.6 %) and fathers (95.2 %) were literate (Table 1).

**Table 1:** Socio-demographic and economic characteristics of school children and their parents in Robe District, southeast Ethiopia, 2015

Characteristics	Frequency	Percent
Sex of the child		
Male	177	45.0
Female	216	55.0
Age of the child (in years)		
6–9	223	46.7
10–12	170	43.3
Religion		
Orthodox	181	46.1
Muslim	198	50.4
Others^a^	14	3.5
Ethnicity		
Oromo	352	89.6
Other^b^	41	10.4
Mother’s education		
Illiterate	37	9.4
Literate	356	90.6
Mother’s employment		
Employed^c^	92	23.4
Housewife	198	50.4
Daily laborer	47	12.0
Merchant	56	14.2
Father’s education		
Illiterate	19	4.8
Literate	374	95.2
Father’s employment		
Employed^c^	103	26.2
Farmer	95	24.2
Daily laborer	58	14.8
Merchant	137	34.8
Household Size		
≤5	110	28.0
>5	283	72.0
Household monthly income (Eth. birr)		
<500	89	22.6
500–1500	98	25.0
≥1500	206	52.4

^a^Protestant and catholic

^b^Amhara, Gurage, Wolayita, Tigre

^c^Governmental and nongovernmental employe

**Fig. 1:**
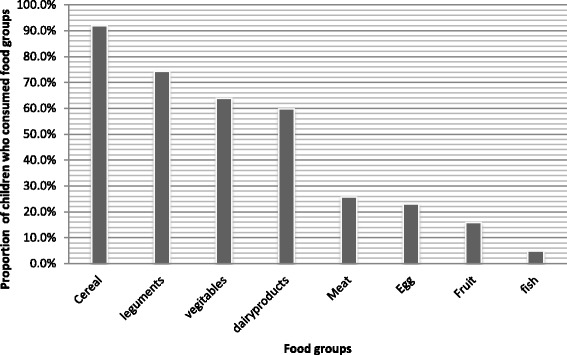
Proportion of school children (6–12 years) consumed food groups in the last 24 h preceding the date of survey, Robe District, Southeast Ethiopia, 2015

The majority (84.2 %) of children had three regular meals per day, while about 75.6 % were served fresh food (Table 2). The dietary pattern of the majority of children were largely based on cereals (91.9 %), and legumes (74.3 %), followed by vegetables (63.9 %) and dairy products (59.9 %). However, the consumption of animal food products was low, in which about one-quarter (25.7, and 23 %, respectively) of children ate meat and egg. Only few, 4.8 %, of them ate fish in the past 24-h (Fig. 1). Almost all (99.7 %) of the parents had private latrine, and accessed water from safe sources (91.7 %). Approximately one-third (28.8 %) of the parents had home gardening for fruit and vegetables (Table 2).

**Table 2:** Feeding pattern, hygiene and sanitation related characteristics of school children in Robe District, southeast Ethiopia, 2015 (*n* =393)

Characteristics	Frequency	Percent
Having regular meals		
Yes	331	84.2
No	62	15.8
Skipping regular meals		
Yes	131	33.3
No	264	66.7
The reasons for skipping regular meals (*n* = 129)		
Shortage of food	61	47.3
Lack of appetite	30	23.3
Sickness	38	29.4
The first served child within a household		
Female	85	21.6
Male	20	5.1
Together	288	73.3
Type of food commonly consumed		
Left over	96	24.4
Fresh	297	75.6
Water treatment habit		
Yes	23	5.9
No	369	93.8
Don’t Know	1	0.3
Method of water treatment (*n* = 23)		
Boiling	14	60.9
Solar disinfection	7	30.4
Others ^a^	2	8.7
Availability of home grading		
Yes	113	28.8
No	280	71.2
Types of food staffs cultivated in the home garden (*n* = 113)		
Fruits	50	44.3
Vegetables	19	16.8
Fruits and vegetables	44	38.9
Purpose of home gardening (*n* = 113)		
For household consumption	72	63.7
For market	4	3.5
For market and household consumption	37	32.8
Availability of latrine		
Yes	392	99.7
No	1	0.3
Type of latrine		
Flush toilet	8	2.0
Pit latrine	192	48.9
Pit latrine without slab	193	49.1

^a^Adding chemical and water agar

In this study, the median urine iodine level was 78 μg/l, and about 57 % [95 % CI: 48.0, 66.0 %] of the children had low urine iodine level (<100 μg/L), suggesting ID. Out of the children with ID, nearly half (42 %) had a urine iodine level of 50–99 μg/l, whereas about 3.3 and 11.7 % had urinary iodine level of <20 μg/l and 20–49 μg/l, respectively.

In addition, the overall prevalence of goiter was 43.5 % [95 % CI: 33.9, 53.1 %], in which about 31.3 and 12.2 % were found with grade 1 and grade 2 goiter, respectively. However, only 29 % of the households had adequately iodized salt. Of those households with inadequately iodized salt (71 %), about 2 and 69 % of the sampled salts were found with ‘0’ppm and ‘<15’ ppm, respectively.

Both the bivariate and multivariate logistic regression analyses were carried out to identify the determinants of ID (urinary iodine level <100 μg/l). Accordingly, the result of the bivariate logistic regression analysis showed that age and sex and educational status of the father were significantly associated with ID. However, in the multivariate logistic regression analysis, age and sex of the child remained significantly and independently associated with ID. Consequently, the odds of ID among children aged 10–12 years were 2.1 times [AOR = 2.1; 95 % CI: 1.44, 3.42] higher as compared to children aged 6–9 years. Likewise, the likelihood of ID among male children was 2.2 times [AOR = 2.2; 95 % CI: 1.54, 3.55] higher than that of female counterparts (Table 3).

**Table 3:** Factors associated with iodine deficiency (urine iodine level <100 μg/l) among school children (6–12 years), Robe District, southeast Ethiopia, 2015 (*n* =393)

Characteristics	Iodine deficiency			
	Yes (#)	No (#)	COR^a^ (95 % CI)	AOR^b^ (95 % CI)
Sex of the child				
Male	80	97	1	1
Female	144	72	2.4 (1.6, 3.5)	2.2 (1.5, 3.6)*
Age of the child (in Years)				
6–9	110	113	1	1
10–12	114	56	2.2 (1.4, 3.4)	1.9 (1.2, 3.1)*
Father’s education				
Illiterate	12	7	1	
Literate	212	162	1.3 (0.6, 8.9)	
Mother’s education				
Illiterate	26	11	1.9 (0.7, 11.1)	
Literate	199	157	1	
Skipping regular meal				
Yes	79	52	1.2 (0.3, 2.4)	
No	145	117	1	
Salt iodine content				
Inadequately iodized	154	125	1	
Adequately iodized	70	44	1.3 (0.8, 3.1)	

*P < 0.05

^a^Crude Odds Ratio

^b^Adjusted Odds Ratio

## Discussion

The median urine iodine concentration of ≥100 ug/l defines a population with no ID [5], however, the present study showed that the median urine iodine concentration among school children was 78 μg/l. The finding was in line with what was reported from India (70 μg/l) [33]. But, it is lower than the WHO/UNICEF/ICCIDD jointly recommended cutoff point (≥100 ug/l) [5] and, reports from India, such as Uttarakhand (125 μg/L) [34] and India (165 μg/L, 235 μg/L) [35]. On the other hand, about 57 % children had low urine iodine level (<100 ug/l) suggesting the presence of ID. This prevalence was in agreement with the report from South Tajiksitan (51.2 %) [36]. The difference in the median urinary iodine level between the current and the former study settings might be related to poor dietary intake of iodine rich food and low utilization of iodized salt in the study area.

This study also demonstrated that utilization of iodized salt (29.3 %) was lower than the WHO recommendation (>90 %) [5], and other African countries, like South Africa (62.4 %), Uganda (96 %), Ghana (75.6 %), and South Sudan (72.9 %) [5]. However, there was a bit improvement as compared to previous local reports, such as the 2011 Ethiopian Demographic and Health Survey Report (13.3 %) [27], Somali Region (7.7 %) [28], and Tigray Region (22.3 %) [20]. This improvement in utilization of iodized salt could be related to the current efforts of the government in implementing the already available programs designed to promote the production and distribution of iodized salt [37–40].

According to WHO criteria, ID is considered as a public health problem if the total goiter rate is >5 % [2]. However in this study setting, the prevalence of goiter was found to be 43.5 %, which confirmed that there is a severe public health problem. Likewise, the current prevalence was higher than the study reports of other developing countries, including Mandy (6.6 %) [25] and Amreli districts, India (25.2 %) [23], Pakistan (10 %) [29], Tanzania (22 %), and Ecuador (30 %) [6].

The prevalence of goiter was lower than previous study reports in Ethiopia; for example Southern Ethiopia (56.2 %) [30], Jimma, southwest Ethiopia (59.1 %) [14], Neksege, northern Ethiopia (71.4 %) [20], and Womberma and Burie, northwest Ethiopia (54 %) [31]. The discrepancy could be attributed to the current improvements in utilization of iodized salt compared to the periods the other studies were conducted.

The result of the adjusted analysis showed the increased odds of ID among children aged 10–12 years compared to children aged 6–8 years. The finding was supported by other studies conducted elsewhere [23, 31–33]. Obviously, iodine requirement increases with age. Because of pubertal growth spurt and intense anabolic period, children aged 10–12 had higher iodine requirements to support their rapid growth as compared to children aged 6–9 years [34]. In addition, this life stage, 10–12 years, is a part of adolescence where 20 % of adult height and 50 % of adult weight are attained [35].

Likewise, the higher odds of ID were observed among female children compared to their male counterparts. This report is in line with previous findings elsewhere [6, 17, 23, 24, 26, 35, 36, 41, 42]. This might be due to a higher iodine requirement of females to support their growth and development [35]. Furthermore, a female hormone (estrogen) has an inhibitory effect on iodine uptake by the thyroid follicular cells [22].

The study showed the magnitude of ID using biochemical and clinical assessment methods, which helps to better understand the problem in the study area. In addition, the utilization of iodized salt was estimated. However, the study is not free from some of limitations. Firstly, only school children were included in this study, where the result may not be generalizable to outschool children. Secondly, hence the study employed a cross-sectional study design, the findings may not show the cause and effect relationship between iodine deficiency and the independent variables.

## Conclusion

In this study, the prevalence of ID (goiter and low urine iodine level) was high, suggesting a severe public health problem. Lower utilization of iodized salt was also documented in the setting. Furthermore, the higher odds of ID was observed among older aged (10–12 years) and female children. Therefore, there is a need to intensify the implementation of universal salt iodization and attention should be given to school children to efficiently address ID.

## Abbreviations

AOR: Adjusted odds ratio
CI: Confidence interval
COR: Crude odds ratio
DHS: Demographic and Health Survey Report
ID: Iodine deficiency
UIL: Urine iodine level
WHO: World Health Organization
